# Iatrogenic Aortic Dissection Presenting With Leg Pain Diagnosed With Point-of-care Ultrasound

**DOI:** 10.5811/cpcem.2019.7.43287

**Published:** 2019-10-14

**Authors:** Matthew Friedman, Armin Gollogly, Enrique Pena, Jennifer Johnson, Tina Dulani

**Affiliations:** * North Shore University Hospital-Northwell Health, Department of Emergency Medicine, Manhasset, New York; † Long Island Jewish Medical Center-Northwell Health, Department of Emergency Medicine, New Hyde Park, New York; ‡ Donald and Barbara Zucker School of Medicine at Hofstra/Northwell, Department of Emergency Medicine, Manhasset, New York

## Abstract

Iatrogenic aortic dissection (IAD) status-post-cardiac catheterization is a rare complication often isolated to the proximal aorta. This is a case of IAD isolated to the distal aorta in a 41-year-old female who presented to the emergency department with right leg pain after undergoing three cardiac catheterizations. The diagnosis of IAD was made upon discovery of an intimal flap in the distal aorta and femoral artery while performing a point-of-care ultrasound to evaluate for deep vein thrombosis.

## INTRODUCTION

Point-of-care ultrasound (POCUS) is a useful diagnostic tool in the emergency department (ED). There is abundant literature supporting the use of ultrasound for the evaluation of undifferentiated patients in the ED.^1^,^2^ This report describes the case of a patient who underwent POCUS to evaluate for a possible deep vein thrombosis (DVT) and was ultimately diagnosed with a distal aortic dissection extending into the right femoral artery. The etiology of this dissection is thought to be iatrogenic secondary to recent cardiac catheterization. Although aortic dissection is a heavily considered diagnosis in the emergency medicine setting, it is a rare complication of cardiac catheterization and subsequently may present with atypical symptoms.

## CASE REPORT

A 41-year-old female with extensive medical history including hypertension, lupus nephritis, anti-phospholipid antibody syndrome, coronary artery disease, and previously treated Libman-Sacks endocarditis presented to the ED with persistent lightheadedness for one week and two days of recurrent nausea and vomiting with decreased oral intake. She had a pertinent surgical history of coronary artery bypass graft and aortic valve replacement secondary to the endocarditis. She was anticoagulated on warfarin and required hemodialysis. The patient also reported two days of right calf pain that occurred only when ambulating. She did not complain of chest pain, back pain, or abdominal pain.

The patient’s initial vital signs included a blood pressure of 171/91 millimeters of mercury, heart rate of 92 beats per minute, respiratory rate of 18 breaths per minute, and oral temperature of 37.1 degrees Celsius. Her oxygen saturation was 99% on room air. On initial evaluation in the ED, the patient appeared in no distress and was alert and oriented to person, place, and time. She answered questions appropriately, and her neurologic examination showed no focal weakness or sensory deficits. Lungs were clear and cardiac exam was noted as regular rate and rhythm without murmur. The patient’s abdomen was soft, non-tender, and non-distended. Her lower extremities were warm and well perfused with normal range of motion and no swelling or calf tenderness. Her peripheral pulses were intact and symmetric bilaterally.

Based on her history and physical examination, the treating physicians were most concerned for an acute viral process or foodborne illness. Nonetheless, given her complaint of right calf pain in the context of a chronic pro-coagulant state, they decided to evaluate for a DVT in the right lower extremity. The patient underwent a POCUS two-point compression examination of the right leg, which showed normal compression of the right femoral and popliteal venous systems. However, an abnormal intraluminal echogenic signal was seen in the right femoral artery, which had the appearance of an intimal flap.

**Image 1:**
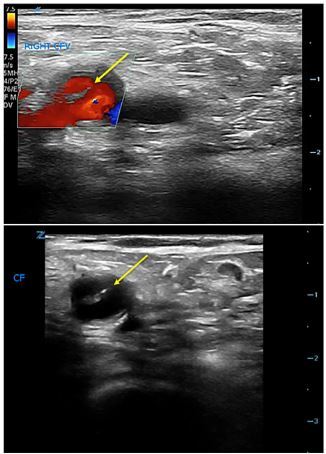
Grayscale and color ultrasound demonstrating intimal flap (arrow) in the right common femoral artery.

**Image 2:**
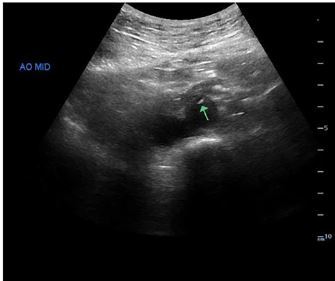
Grayscale ultrasound demonstrating intimal flap (arrow) in mid-aorta.

**Image 3:**
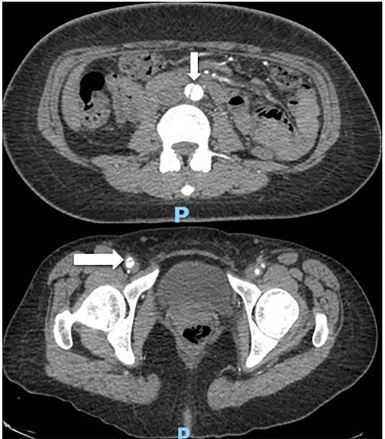
Computed tomography angiogram demonstrating flap in mid-aorta (top arrow) and flap in right common femoral artery (bottom arrow).

Color Doppler was used to confirm differential flow on either side of the flap (Image 1). The ultrasonographers proceeded to interrogate the abdominal aorta, and a dissection flap was noted in the transverse view (Image 2). A computed tomography (CT) angiogram of the chest, abdomen, and pelvis with run-off to the lower extremities was then performed, which showed an intimal flap starting in the distal abdominal aorta and extending into the right common iliac, external iliac, and superficial femoral arteries (Image 3).

A subsequent review of the patient’s medical chart showed that she had been admitted to our institution one month prior for acute coronary syndrome and had been taken to the cardiac catheterization suite three times during that hospitalization. The hospital record noted that she was canalized in her femoral region three times, twice via her left femoral artery and once via her right femoral artery. The patient was assessed by the vascular surgery team in the ED. Their impression was this dissection was iatrogenic given her history of recent catheterization, and they recommended strict blood pressure control and admission. Given her extensive and complicated cardiovascular history she was ultimately admitted to the cardiac intensive care unit. Her blood pressure medications were adjusted, and she was discharged home three days later.

**CPC-EM Capsule**

What do we already know about this clinical entity?

*Post-cardiac catheterization aortic dissection (AD) is a rare but known complication*.

What makes this presentation of disease reportable?

*To our knowledge, this is the first report of a post-cardiac catheterization aortic dissection isolated to the abdominal aorta and distal arteries*.

What is the major learning point?

*Iatrogenic AD is a rare but important complication of cardiac catheterization. Clinical astuteness during ultrasound evaluation is paramount*.

How might this improve emergency medicine practice?

*Consideration of iatrogenic AD in post-catheterization patients presenting with vague symptoms can lead to earlier diagnosis and treatment*.

## DISCUSSION

The advantage of POCUS is that image acquisition and interpretation are performed in real time by clinicians who can quickly integrate unexpected findings into the overall clinical picture. In this case, we were able to reorient from performing a DVT study to fully investigating what appeared to be a femoral artery dissection. By tracing the intimal flap proximally to the abdominal aorta, we rapidly diagnosed the patient’s aortic dissection and promptly initiated tight blood pressure control. A CT angiogram was then performed to define the extent of the dissection. Without POCUS, this diagnosis may have been delayed or missed. This case highlights the importance of remaining astute during the clinical evaluation of a complicated patient.

When aortic dissection is diagnosed using POCUS, the most common scenario is a proximal aortic dissection associated with aneurysmal dilation of the aortic root.^3^ Our case is unusual among reports of aortic dissection identified on ultrasound because the diagnosis was made by finding an intimal flap in the distal aorta and femoral artery. Aortic dissection after coronary angiography is a known complication, but it is rare. In a review by the Registry of Aortic Iatrogenic Dissection of 108,083 catheterization procedures that occurred from 2000–2014, the incidence of aortic dissection was 0.062%.^5^ More recent, smaller case series report similar incidences.^6^,^7^ Of note, these were all ascending dissections. To our knowledge, there are no published reports of iatrogenic aortic dissections (IAD) after coronary angiography isolated to the abdominal aorta and distal vasculature.

Etiology of IAD is suspected to be related to wire or catheter trauma, and there is suggestion that vessels with greater calcification are more prone to IAD.^10^ In general, there are no guidelines for managing IAD. IAD has been successfully managed surgically with stenting, as well as conservatively.^7^ In the available cases series, none of the IAD were of the abdominal aorta. In general, Stanford B or abdominal aortic dissections are managed either medically or with endovascular stenting.^8^,^9^ Factors influencing more aggressive treatment include hemodynamic instability, false lumen expansion, and failure of medical management. Our patient was successfully treated with medical management alone.

Diagnosis of aortic dissection can be made by CT, magnetic resonance imaging, and ultrasound. Studies done to ascertain the sensitivity and specificity of ultrasound in the diagnosis of aortic dissection show wide variability from a sensitivity of 52–80% and a specificity from 0–100%.^13^,^15^ The presence of an intimal flap, however, was found to be 100% specific and 67% sensitive for aortic dissection.^14^,^15^ Although ultrasound should never be the definitive test for aortic dissection, as it lacks sufficient sensitivity, the presence of an intimal flap is highly specific.^4^ Moreover, using POCUS to evaluate suspected aortic dissection has been shown to greatly reduce mean time to diagnosis, which can be life- and limb-saving with such a time-sensitive disease process.^3^ Our patient had claudication symptoms, which could have progressed to frank leg ischemia if the dissection had worsened.

## CONCLUSION

Iatrogenic aortic dissection after coronary angiography is a rare diagnosis, and this case is the first report to our knowledge of a dissection isolated to the distal aorta and its branches. Remaining alert to the possible complication of post-catheterization aortic dissection is important for the emergency physician. This case also reinforces the importance of promoting and maintaining strong bedside sonographic skills within emergency medicine. Our patient was found to have fully compressible veins, but the recognition of a dissection flap in the femoral artery was a critical finding that could have been missed without a well-grounded and methodical approach.

## Supplementary Information

Video. Grayscale ultrasound demonstrating intimal flap in the right common femoral artery.
